# Multi-State Energy Classifier to Evaluate the Performance of the NILM Algorithm

**DOI:** 10.3390/s19235236

**Published:** 2019-11-28

**Authors:** Sanket Desai, Rabei Alhadad, Abdun Mahmood, Naveen Chilamkurti, Seungmin Rho

**Affiliations:** 1 Department of Computer Science and Information Technology, La Trobe University, Melbourne 3086, Australia; s6desai@students.latrobe.edu.au (S.D.); R.Alhadad@latrobe.edu.au (R.A.); A.Mahmood@latrobe.edu.au (A.M.); N.Chilamkurti@latrobe.edu.au (N.C.); 2 Department of Software, Sejong University, Seoul 05006, Korea

**Keywords:** non-intrusive load monitoring, smart grid, smart metering, performance metrics, privacy, energy disaggregation, data collection

## Abstract

With the large-scale deployment of smart meters worldwide, research in non-intrusive load monitoring (NILM) has seen a significant rise due to its dual use of real-time monitoring of end-user appliances and user-centric feedback of power consumption usage. NILM is a technique for estimating the state and the power consumption of an individual appliance in a consumer’s premise using a single point of measurement device such as a smart meter. Although there are several existing NILM techniques, there is no meaningful and accurate metric to evaluate these NILM techniques for multi-state devices such as the fridge, heat pump, etc. In this paper, we demonstrate the inadequacy of the existing metrics and propose a new metric that combines both event classification and energy estimation of an operational state to give a more realistic and accurate evaluation of the performance of the existing NILM techniques. In particular, we use unsupervised clustering techniques to identify the operational states of the device from a labeled dataset to compute a penalty threshold for predictions that are too far away from the ground truth. Our work includes experimental evaluation of the state-of-the-art NILM techniques on widely used datasets of power consumption data measured in a real-world environment.

## 1. Introduction

Recent social advancements and rapid industrialization have led to concerns about climate change and the ever-increasing demand for energy, which is a recognized problem of international significance. The World Energy Outlook Report [1] indicates that global energy demand is set to grow by 90% by 2040. The need for the efficient use of energy resources and reduced carbon footprints has led to a systematic deployment of cyber–physical systems (CPS) such as smart grid [2]. A smart grid enables the distribution and consumption of energy resources in a more efficient, effective and economical way. Smart meters are now an integral part of advanced metering infrastructure (AMI) of a smart grid that allows appliance load monitoring (ALM) [3] to enable real-time energy consumption reporting and feedback.

Non-intrusive load monitoring (NILM) is a process of estimating the energy consumption of the appliances in a consumer’s (e.g., household or industry) premises. NILM is a non-intrusive technique that estimates appliance-level energy consumption based on the aggregated power consumption readings gathered from a consumer’s smart meter [3]. NILM also enables real-time monitoring and feedback on the end-user’s appliance consumption. It also allows utilities to perform real-time load analysis and more accurate energy forecasting, which saves them operational time and expense. This feedback gives the consumer insight into the amount of energy an appliance consumes to help make informed decisions about conserving power, whether motivated by economic or ecologic concerns (or both). Research findings suggest that residential appliance-level power usage feedback results in savings of up to 12% of annual power consumption [4]. Feedback also improves awareness of one’s behavior. The more closely electricity consumption can be linked to specific appliances and activities, the clearer the relevance of the behavior becomes. Detailed appliance-specific feedback i.e., the operational state can help a consumer determine as to how a certain appliance behaves and its effect on electricity consumption whether economic or ecological. This also increases the sense of control because the consumer can find out how changes in behavior or appliance operation can affect the outcome [5].

Research in NILM has made advances in integrating a combination of signal processing, statistical and machine learning technologies to provide a cost-effective approach for load forecasting [6], real-time monitoring, and feedback [7]. However, one of the key issues is to accurately evaluate and report the performance of existing NILM approaches. Recent research findings [8,9] on NILM algorithms and their implementation conclude that there are some practical limitations of the existing metrics: first, existing event classification metrics do not classify multi-state devices accurately with respect to events in the original ground truth; second, although the overall energy of a device is estimated, it does not measure the energy estimation of each classified state of the device; finally, with relatively large errors the metric result exceeds the usual accuracy interval of 0 and 1, making it less intuitive and explainable.

This paper solves these problems by proposing multi-state energy classifier (MEC) which is a new metric based on unsupervised clustering technique that combines event classification and energy estimation by identifying the operational states of the device from a labeled dataset to compute a penalty threshold for predictions that are too far away from the ground truth. We evaluate our approach using the widely accepted NILMKTK [10] framework and various publicly available datasets such as the Reference Energy Disaggregation dataset (REDD) [11], Dutch Residential Energy dataset (DRED) [12] and Almanac of Minutely Power dataset (AMPds) [13].

### 1.1. Motivation and Related Works

**Figure 1:**
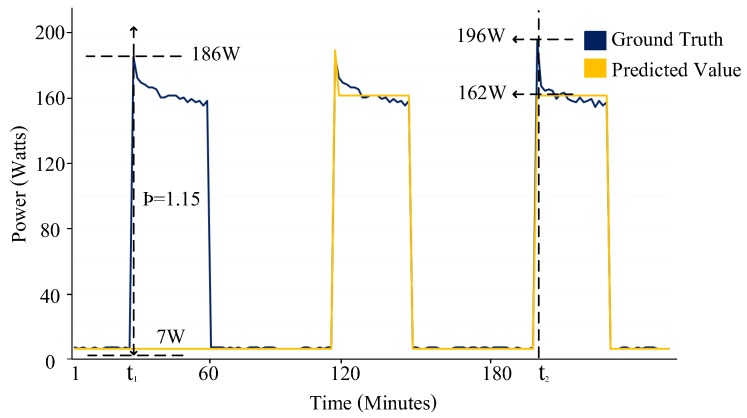
Power signal pattern of a type-IV (always on) appliance fridge.

NILM takes the aggregate power readings from a smart meter and predicts power levels and device states for every appliance connected to the smart meter. Figure 1 presents the ground truth power signal pattern (blue) and the disaggregated output (yellow) of a NILM algorithm for the fridge. Although NILM techniques have been applied widely for real-time monitoring and energy consumption feedback, the accurate evaluation of NILM approaches has been a critical issue, especially for multi-state devices. An accurate evaluation of different operational states of a multi-state device can help the consumer gain valuable insight as to how a certain appliance behaves, its operational efficiency and the effect on electricity consumption. Several performance metrics have been proposed and used by researchers to evaluate NILM algorithms.

Tsai et al. [14] and Chang et al. [15] used the concept of recognition accuracy, which works at a very high sampling rate (e.g., 1 μs to 100 ms) to match patterns. However, these techniques cannot be directly applied to smart-meter-based power disaggregation since smart meters report data at a much lower sampling rate (e.g., 1 s up to 10 min based on utility settings). Batra et al. [16] used root mean square error (RMSE) as one of the energy estimation accuracy metrics. RMSE measures how spread out the predicted values are from their ground truth. The measure is not normalized which makes it difficult to compare the disaggregation accuracy between different appliances. The normalized disaggregation error (NDE) [17] metric addresses the normalization issue of RMSE. However, NDE tends to report inflated accuracy.

Kolter et al. [11] proposed total energy correctly assigned (TECA), a method to report estimation accuracies. However, the metric tends to report inflated accuracies. As shown in Figure 1, a fridge has the ground-truth value of 186W (compressor ON-state) and an estimated value of 7W (compressor off-state) for a given time period $t_{1}$. The TECA metric reported accuracy of 51% for time $t_{1}$. Huang et al. [18] and Osathanunkul et al. [19] used the information retrieval domain metric F1-score to evaluate the performance of the energy disaggregation approaches for different sampling rates. The information retrieval domain metric F-score does not differentiate between the multiple operational states of an appliance.

Kim et al. [20] presented a modified F-score (M-Fscore) which combines the appliance state classification and power estimation accuracies together. The MF-score applies a threshold of standard deviation by the mean to divide the true positive (TP) into accurate true positive (ATP) and inaccurate true positive (ITP) for the appliance. However, the MF-score does not consider the multistate characteristic of an appliance.

As shown in Figure 1, suppose we have an appliance (fridge) with σ of 82.31 and a μ of 70.99, then the threshold ρ is 1.15. For a given time period $t_{1}$, the ground truth value of the fridge is 186 W (compressor on-state) and the estimated value is 7 W (compressor off-state). The higher threshold ρ resulted in classifying this event as an ATP which would result in an inaccurate increase in reporting NILM accuracy.

Makonin et al. [21] proposed Finite-state F-score (FS-FScore) to calculate the accuracy of a non-binary classification. A partial penalization measure called an inaccurate portion of true-positive (inacc) was introduced to convert the binary nature of TP into a discrete measure. There are two problems associated with FS-Score. First, the calculation of inacc requires the knowledge of pre-defined states of an appliance. Second, while the FS F-score differentiates between multiple states, it does not correctly consider the measurement variations within the same operational state. For example, for a given time period $t_{2}$ in Figure 1, the ground truth value of the fridge is 196 W and the estimated value is 162 W. Clearly, the metric does not penalize the algorithm for such a large variation.

### 1.2. Contribution

In this paper, we propose a novel performance evaluation metric multi-state energy classifier (MEC) which can be used to accurately measure the performance of the NILM algorithms, yielding the following contributions:

- the proposed metric accurately classifies the operational states of an appliance of different categories with respect to events in the original ground truth;
- the proposed metric combines energy estimation with event classification to accurately quantify and penalize the algorithm with respect to variation in the measurements of the state of an appliance;
- evaluation and implementation of two state-of-the-art NILM approaches and their performance with several existing and proposed evaluation metrics (see Section 4);

The paper is organized as follows. In Section 2, we briefly discuss the technological concepts used in this work. In Section 3, we present the proposed metric and perform classification and estimation testing in Section 4 on real-world publicly available datasets. We look at why researchers need to report accuracy with respect to both event classification and energy estimation and conclude the paper in Section 5.

## 2. Background

### 2.1. Energy Disaggregation

The energy disaggregation problem can be formulated as follows: given a smart meter SM, there exists an aggregate power consumption series $P=\{p_{1},p_{2},p_{3},\ldots ,p_{t}\}$ for time T={1,2,3,….,t}, we want to infer the power consumption $y_{t}^{i}$ of appliance i∈{1,2,3,…,M} of the M active appliances, such that

(1) $$P_{T}=\sum_{i=1}^{M}y_{t}^{\left(i\right)}+\sigma \left(t\right)$$

where σ(t) represents unaccounted power or noise.

**Figure 2:**
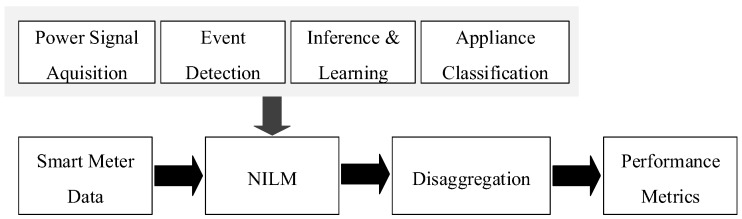
Non-intrusive load monitoring (NILM) process.

A NILM system consists of four steps as shown in Figure 2: *power signal acquisition and pre-processing*; *event detection and feature extraction*; *inference and learning*; and *appliance classification*. Power signal acquisition is the first step in energy disaggregation and is responsible for acquiring aggregated load measurements at a different sampling rate. The Event Detection and Feature Extraction step involves noting down the steady-state or transient state changes in these pre-processed power measurements. Features corresponding to these events are extracted. These are unique consumption patterns corresponding to each individual appliance operation. In the Learning and Inference step, the necessary supervised or unsupervised methods are applied to determine the appliances. The final step, appliance classification involves dividing the total aggregate readings into individual appliance states and the power consumption corresponding to that appliance state [22].

### 2.2. Appliance States

NILM enables the identification of individual appliances with their operating states and the corresponding power consumption. An appliance can operate in different states as per their functionality or use. Researchers have presented four abstract models, commonly used to represent and categorize these appliances [23]:

- Type 1—on/off appliances: On/off type of devices have a pre-defined set of operation, i.e., two states of either being on or off at a given time period. This category represents various basic home appliances such as toaster, light bulb, water pump, etc. The On state corresponds to a specific amount of power.
- Type II—finite state machines or multi-state appliances: Multi-state appliances consist of more than one state of operation i.e., active state. Each active state or the operational state has a corresponding energy consumption. This category includes devices such as washing machines, stove burners, fridges, etc.
- Type III—infinite state or continuously variable appliances: Continuously variable appliances do not have a finite set of states. Such appliances are a challenge in relation to the concept of energy disaggregation as they are difficult to model or identify using NILM algorithms. An infinite state includes appliances such as light dimmers, power drills, battery chargers, laptops, phones, etc.
- Type IV—always on: the type IV category refers to appliances that have a constant source of consumption. These appliances may have single or multiple operational states. The appliances in this category include smoke alarms, fridges, landline phones, etc.

**Figure 3:**
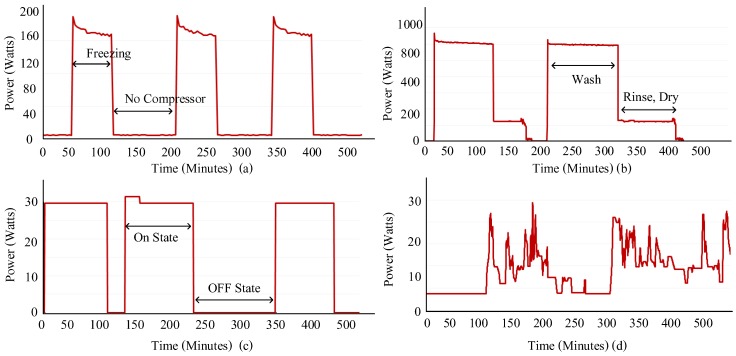
Power pattern of devices in the aforementioned appliance categories (**a**) type IV (always on): fridge (**b**) type II (multi-state): clothes washer (**c**) type I (on/off): fan (**d**) type III (infinite state): laptop.

These operating states of a device are very important to accurately classify the appliances and also estimate their power consumption [3]. Figure 3 shows the power patterns of different appliances in the aforementioned categories.

### 2.3. NILM Dataset

A NILM dataset is a publicly available dataset consisting of power consumption data measured in a real-world environment such as a house or a building. It consists of smart metering (i.e., aggregate) data and may or may not have an individual device (i.e., ground truth) power consumption data based on the purpose of the dataset. To evaluate the performance of NILM algorithms, it is essential to have the ground truth for an appliance for which the disaggregation is being performed. In this paper, we use the three most widely used datasets: Reference Energy Disaggregation dataset (REDD), Dutch Residential Energy dataset (DRED) and Almanac of Minutely Power dataset (AMPds) for energy disaggregation.

### 2.4. Unsupervised Clustering

Clustering refers to unsupervised learning algorithms that do not need pre-labeled data to extract rules for grouping similar data instances [24]. Based on different criteria, a clustering process partitions the data differently. There are various types of clustering techniques, however, we will discuss the one that has been used in the proposed metric.

#### Basic K-Means Algorithm

The k-means algorithm is a well-known unsupervised partitioning algorithm. The k-means is a point-based clustering method that allocates a data point to the most similar cluster and updates the center value of the cluster. This process is done iteratively until the cluster assignment is stable. The k-means cluster is represented by the mean value of the data points in that cluster, also known as the centroid. The distance between each data point of the cluster and mean value i.e. the centroid is measured using Euclidean’s distance. The k-means algorithm is a widely used partition algorithm based on determining the number of groups by defining the initial centroid value. However, it requires the user to provide the number of clusters (k) [25]. An approach for providing the value of k is to use the Elbow method.

### 2.5. Performance Metrics in NILM

Performance metrics are one of the evaluation standards which enable empirical evaluation and comparison of different NILM approaches. One of the most basic accuracy measures is defined as

(2) $$Acc=\frac{CorrectMatches}{CorrectMatches+IncorrectMatches},$$

where *CorrectMatches* refer to a correct prediction by a NILM technique based on the ground truth and the predicted values.

**Figure 4:**
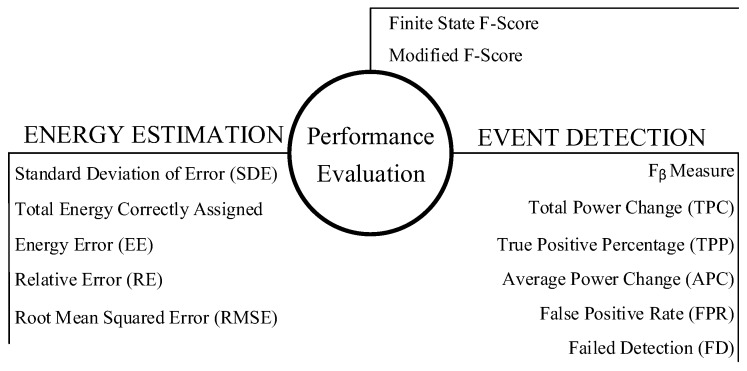
Performance evaluation metrics for energy disaggregation.

NILM researchers have also used several performance metrics to evaluate energy disaggregation divided into categories as shown in Figure 4.

#### 2.5.1. Standard Metrics

Event detection metrics were designed to keep track of the energy usage patterns of the consumer over time. Event detection metrics enable NILM to keep track of individual events and usage patterns over a given time period. Event detection metrics consist of metrics such as F-measure, total correctly assigned energy (TECA) and accuracy (A).

Energy estimation metrics were designed to compare and evaluate the NILM disaggregation results i.e., predicted results versus the actual energy consumption i.e., ground truth. Energy estimation metrics consist of probabilistic techniques such as root mean square error (RMSE), R squared, mean average error (MAE), etc. which show how far the predictions are from the actual results.

#### 2.5.2. State-of-the-Art Metrics

State-of-the-art metrics were proposed by researchers to measure the accuracy of NILM algorithms by combining event classification and power estimation.

- Modified F-score (M-FScore): modified F-score [20] is a modified version of F-score to account for non-binary outcomes, such as a power signal. The metric splits the True positive into accurate true positive (ATP) and incorrect true positive (ITP). A threshold **T** was introduced to divide the true positive (TP) into ATP and ITP. The threshold **T** is calculated by dividing the standard deviation by the mean of the whole ground truth of an appliance.
  $g_{t}>0$ and $p_{t}>0$, $\frac{|p_{t}-g_{t}|}{g_{t}}\leq$ **T**, then the prediction is ATP
  $g_{t}>0$ and $p_{t}>0$, $\frac{|p_{t}-g_{t}|}{g_{t}}>$ **T**, then the prediction is ITP
  The ATP and ITP are applied to the Precision and Recall while the definition of F-score remains the same.
- Finite state F-score (FS F-score): finite state F-score [21] converts the binary nature of the TP into a discrete measure by introducing a partial penalty *inacc*. The *inacc* is defined as
  (3) $$inacc=\sum_{t=1}^{T}\frac{|p_{t}^{\left(m\right)}-g_{t}^{\left(m\right)}|}{K^{\left(m\right)}},$$
  where $p_{t}^{\left(m\right)}$ is the estimated state of appliance m at time t, $g_{t}^{\left(m\right)}$ is the ground truth state, and $K^{\left(m\right)}$ is the number of states for appliance *m*. The inacc is applied to the precision and recall while the definition of F-score remains the same.

## 3. Proposed Metric

**Figure 5:**
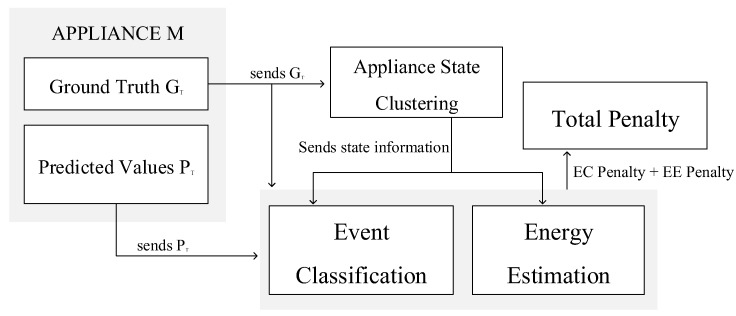
Multi-state energy classifier (MEC) overview.

This section presents the MEC metric, as shown in Figure 5. Figure 5 illustrates the overall MEC process which comprises three important steps: appliance state clustering; event classification penalty; and energy estimation penalty.

Algorithm 1 describes the process depicted in Figure 5. Line 1 of Algorithm 1 identifies the operational states of the appliance. The operational states compute the required parameters and the threshold to accurately penalize misclassification or incorrect energy estimation. We apply the penalty for inaccurate event classification in line 2. Next, we penalize the incorrect energy estimation in line 3. The total penalty for incorrect event classification and inaccurate energy estimation is computed in line 4.

**Algorithm 1** Multi-state energy classifier (MEC).**Input**: $G_{T}=\left\{g_{1},g_{2}\ldots g_{t}\right\}$ is the ground truth of appliance *m*
$P_{T}=\left\{p_{1},p_{2}\ldots p_{t}\right\}$ is the predicted values of appliance *m*
ε = Accuracy weightage for event classification
(1−ε) = Accuracy weightage for energy estimation
**Output**:
MEC = MEC accuracy for appliance *m*
1:$C_{State}$ = $ApplianceStateClustering(G_{T})$2:$Penalty_{EC}^{\left(m\right)}$ = ECPenalty ($G_{T}$,$P_{T}$,$C_{State}$)3:$Penalty_{EE}^{\left(m\right)}$ = EEPenaly ($G_{T}$,$P_{T}$,$C_{State}$)4:$TotalPenalty_{m}$ = $\left(\varepsilon \cdot Penalty_{EC}^{\left(m\right)}\right)$ + $\left(\left(1-\varepsilon \right)\cdot Penalty_{EE}^{\left(m\right)}\right)$5:**return**$TotalPenalty_{m}$

(4) $$TotalPenalty_{m}=\left(\varepsilon \cdot Penalty_{EC}^{\left(m\right)}\right)+\left(\left(1-\varepsilon \right)\cdot Penalty_{EE}^{\left(m\right)}\right)$$

**Figure 6:**
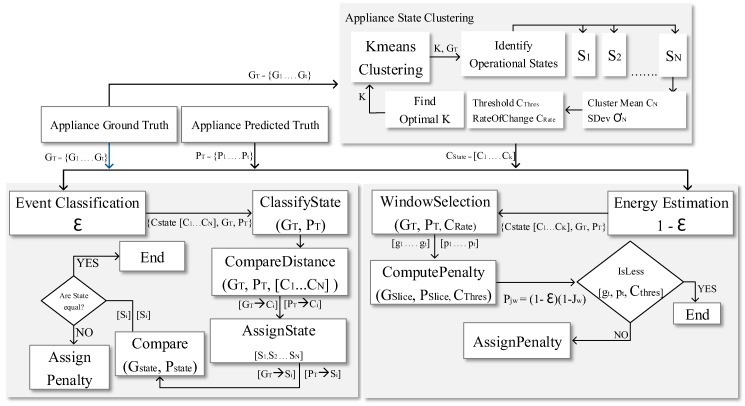
Detailed process of multi-state energy classifier (MEC).

The total penalty is divided into two parts: event classification penalty and energy estimation penalty. A user-supplied parameter ϵ enables the users to assign more or less weight to either type of penalty according to their requirement. The total penalty is the weighted sum of the individual penalties (Equation (4)). The three key processes of the MEC metric are presented in detail in the following subsections and also presented in Figure 6.

### 3.1. Appliance State Clustering

The appliance state clustering process identifies different clusters that relate to the different operational states of an appliance. To improve the performance of event classification and energy estimation, the usage of the clustering scheme is an important factor. In this paper, we use the k-means algorithm for clustering the operational states of the appliance based on the ground truth data available in the NILM dataset.

To determine the number of clusters, we use the elbow method with k-means clustering.

Once the number of clusters is determined, the k-means clustering algorithm is applied to the appliance ground truth. Based on the unlabelled clustering results, we identify the different operational states of an appliance. Furthermore, we compute the parameters related to the operational state of the appliances as shown in Algorithm 2 which will be used by Algorithms 3 and 4.

**Algorithm 2** Appliance state clustering.**Input**: $G_{T}=\left\{g_{1},g_{2},\ldots ,g_{t}\right\}$ is the ground truth of appliance *m*
*N* = Maximum number of states
**Output**:
$C_{State}=\left[C_{1},C_{2},\ldots ,C_{K}\right]$ is the clustered operational states of appliance *m*
  1:$G_{Scale}=$ Standardize the values of $G_{T}$ time series  2:**for**K=1**to***N***do**  3:  Compute within groups sum of squares (WSS)  4:**end for**  5:Obtain *K* using elbow method  6:Perform K-Means clustering on $G_{T}$ to find *K* clusters $Clus_{1},Clus_{2},\ldots ,Clus_{K}$  7:where $Clus_{1}=\left\{G_{T\_Clus_{1}}^{\left(1\right)},\ldots ,G_{T\_Clus_{1}}^{\left(n_{1}\right)}\right\},\ldots ,Clus_{K}=\left\{G_{T\_Clus_{K}}^{\left(1\right)},\ldots ,G_{T\_Clus_{K}}^{\left(n_{K}\right)}\right\}$  8:**for**i=1**to***K***do**  9:  Get $C_{mean}$ and $C_{std.dev}$ of cluster $Clus_{i}$10:  $C_{Thres}=\frac{C_{std.dev}}{C_{mean}}$11:  $C_{Rate}=\frac{\lambda C_{std.dev}}{C_{mean}}$ where λ=312:  Store $\left[S_{i},C_{mean},C_{std.dev},C_{Thres},C_{Rate}\right]$ in $C_{i}$13:  Store $C_{i}$ in $C_{State}$14:**end for**15:**return**$C_{state}$

### 3.2. Event Classification Penalty

As explained in Section 1.1, the existing metrics often overestimate the accuracy of a NILM algorithm due to the incorrect classification of multiple states of an appliance. Algorithm 3 quantifies the inaccuracy of an event that has been misclassified by the NILM algorithm and applies a penalty based on the appliance states computed in Algorithm 2. Algorithm 3 describes the process depicted in Figure 6 in detail.

**Algorithm 3** Event classification penalty (ECPenalty).**Input**: $G_{T}=\left\{g_{1},g_{2}\ldots g_{t}\right\}$ is the ground truth of appliance *m*
$P_{T}=\left\{p_{1},p_{2}\ldots p_{t}\right\}$ is the predicted values of appliance *m*
$C_{State}$ is the clustered operational state data of appliance *m*
**Output**:
$\sum_{i=1}^{T}EC_{i}^{\left(m\right)}$ is the total Event Classification Penalty for appliance *m*
  1:Set $TotalPenalty_{m}=0$  2:**for**t=1**to***T***do**  3:  Get datapoint $g_{t}$ and $p_{t}$  4:  **if** $g_{t}>0$ **and** $p_{t}>0$ **then**  5:    Compute closestCluster $\left(g_{t},C_{State}\left[C_{i\_C_{mean}}\right]\right)$  6:    Set state of $g_{t}$ to $C_{i\_S_{i}}$  7:    Compute closestCluster $\left(p_{t},C_{State}\left[C_{i\_C_{mean}}\right]\right)$  8:    Set state of $p_{t}$ to $C_{i\_S_{i}}$  9:  **end if**10:  **if** state of $g_{t}\neq p_{t}$ **then**11:    Set penalty $EC_{t}^{\left(m\right)}$ equal to 112:  **else**13:    Set penalty $EC_{t}^{\left(m\right)}$ equal to 014:  **end if**15:**end for**16:**return**$\sum_{i=1}^{T}EC_{i}^{\left(m\right)}$

The input for Algorithm 3 is the operational states information $C_{State}$ (output from Algorithm 2), the ground truth $G_{T}=\left\{g_{1},g_{2}\ldots g_{t}\right\}$ and the predicted values $P_{T}=\left\{p_{1},p_{2}\ldots p_{t}\right\}$ of a NILM algorithm for appliance *m*. Next, in Line 3, Algorithm 3 takes the data points $(g_{i}$, $p_{i})$ that correspond to the TP output from an NILM algorithm. For a True Positive prediction of a NILM, $g_{i}$ refers to the ground truth value while $p_{i}$ refers to its corresponding predicted value. Lines 5–8 obtain the clusters (obtained from Algorithm 2) closest to the data points ($g_{i}$, $p_{i}$) and matches the states $S_{i}$ of the assigned clusters in Lines 6 and 8. Lines 10–14 assign a penalty $EC_{t}^{\left(m\right)}$ if the states of corresponding data points $(g_{i}$, $p_{i})$ do not match. We define $\sum_{i=1}^{T}EC_{i}^{\left(m\right)}$ as the total penalty for the inaccurate classification of operational state. The energy estimation penalty is explained next.

### 3.3. Energy Estimation Penalty

The energy estimation penalty process quantifies the inaccuracy of the estimated energy using an NILM algorithm. Algorithm 4 describes the process depicted in Figure 6 in detail.

**Algorithm 4** Energy estimation penalty (EEPenalty)**Input**: $G_{T}=\left\{g_{1},g_{2}\ldots g_{t}\right\}$ is the ground truth of appliance *m*
$P_{T}=\left\{p_{1},p_{2}\ldots p_{t}\right\}$ is the predicted values of appliance *m*
$C_{State}$ is the clustered operational state data of appliance *m*
$G_{Slice}$ is a vector of all IR
$P_{Slice}$ is a vector of all IR
**Output**:
$\sum_{i=1}^{T}EE_{i}^{\left(m\right)}$ is the total Energy estimation Penalty for appliance *m*
  1:Init k,l=1  2:**for**t=1**to***T***do**  3:  Obtain data point $g_{t}$ and $p_{t}$  4:  **if** $g_{t}>0$ **and** $p_{t}>0$ **then**  5:    Compute $C_{g}=$ closestCluster $\left(C_{State},g_{t}\right)$  6:    Compute $C_{p}=$ closestCluster $\left(C_{State},p_{t}\right)$  7:    Obtain $C_{g\_C_{Rate}}$ and $C_{p\_C_{Rate}}$  8:    Set k=t and l=t  9:    **while** ($\left(\frac{|g_{t+1}-g_{t}|}{g_{t}}<C_{g\_C_{Rate}}\right)$ **and** $\left(\frac{|p_{t+1}-p_{t}|}{p_{t}}<C_{p\_C_{Rate}}\right)$) **do**10:      Add data point $g_{t}$ to $G_{Slice}$11:      Add data point $p_{t}$ to $P_{Slice}$12:      Increment *l*13:    **end while**14:    Set t=l+115:    $P_{jw}=$ Call ComputePenalty($G_{Slice},P_{Slice}$)16:    Call AssignPenalty($G_{T},P_{T},P_{jw}$)17:  **end if**18:**end for**19:**return**$\sum_{i=1}^{T}EE_{i}^{\left(M\right)}$20: 21:**Procedure** ComputePenalty $\left(G_{Slice},P_{Slice}\right)$22:Compute $J_{w}\left(G_{Slice},P_{Slice}\right)=\frac{\sum_{i}min\left(g_{i},p_{i}\right)}{\sum_{i}max\left(g_{i},p_{i}\right)}$23:Compute $P_{jw}=\left(1-J_{w}\left(G_{Slice},P_{Slice}\right)\right)$24:**EndProcedure**25: 26:**Procedure** AssignPenalty $\left(G_{T},P_{T},P_{jw}\right)$27:**for**i=k**to***l***do**28:  **if** $\left(\left(g_{i}>0\right)\mathrm{and}\left(p_{i}>0\right)\mathrm{and}\left(\frac{|p_{i}-g_{i}|}{g_{i}}>C_{g\_C_{Thres}}\right)\right)$ **then**29:    Assign penalty $EE_{i}^{\left(m\right)}=P_{jw}$30:  **else**31:    Assign penalty $EE_{i}^{\left(m\right)}=0$32:  **end if**33:**end for**34:**EndProcedure**

Algorithm 4 takes the operational states information $C_{State}$ (output from Algorithm 2), the ground truth and the predicted values of an appliance as an input to provide a penalty for an inaccurate estimation $EE_{i}^{\left(m\right)}$. Similar to the event classification penalty process, we implement Algorithm 4 for all the predicted TP values from an NILM algorithm. The energy estimation penalty process is subdivided into three steps:

Step 1—window selection: in the window selection process, the basic idea of Algorithm 4 is to divide the time series values of ground truth $G_{T}$ and the corresponding predicted values $P_{T}$ into windows, based on changes in the power consumption that reflect a change in the operational state of an appliance as shown in Figure 9. The algorithm starts by traversing through the data points of the ground truth time series $G_{T}=\left\{g_{1},g_{2}\ldots g_{t}\right\}$ and the predicted value time series $P_{T}=\left\{p_{1},p_{2}\ldots p_{t}\right\}$. The operational states of the starting data points $g_{t}>0$ and $p_{t}>0$ are determined by assigning the data points to their closest clusters $C_{g}$ and $C_{p}$ for the ground truth and the predicted values, respectively. Next, to check if the following points i.e., $g_{t+1}$ and $p_{t+1}$ belong to the same state, line 10 checks the rate of change of power using $\frac{|g_{\left(t+1\right)}-g_{t}|}{g_{t}}<C_{g\_C_{Rate}}$ and $\frac{|p_{\left(t+1\right)}-p_{t}|}{p_{t}}<C_{p\_C_{Rate}}$. The $C_{g\_C_{Rate}}$ and $C_{p\_C_{Rate}}$ are thresholds for the clusters to which $g_{t}$ and $p_{t}$ belong to. The threshold $C_{Rate}$ is defined as $C_{Rate}=\frac{\lambda C_{std.dev}}{C_{Mean}}$, where λ=3 represents 99.7% probability that the points belong to that cluster.

While traversing through the time series, if Algorithm 4 detects a rate of change in either of the time series $G_{t}$ and $P_{t}$, it marks the end of the same operational state and stores them in $G_{Slice}$ and $P_{Slice}$ respectively (lines 10–11). The traversing process in this step ensures that; firstly $G_{Slice}$ and $P_{Slice}$ only contain true positives; secondly, the data points in $G_{Slice}$ and $P_{Slice}$ belong to the same operational state as their members respectively.

Step 2—computing energy estimation penalty: the next step in Algorithm 4 involves calculating the penalty for the $G_{Slice}$ and $P_{Slice}$. In line 15, Algorithm 4 calls the $ComputePenalty(G_{Slice},P_{Slice})$ procedure defined in Line 21–26. Next, the ComputePenalty() procedure calculates the penalty $P_{jw}=\left(1-J_{w}\left(G_{Slice},P_{Slice}\right)\right)$ in Line 24, where $J_{w}\left(G_{Slice},P_{Slice}\right)$ is $\frac{\sum_{i}min\left(g_{i},p_{i}\right)}{\sum_{i}max\left(g_{i},p_{i}\right)}$.

Step 3—assigning energy estimation penalty: the third step of Algorithm 4 is to assign the penalty computed in the previous step. In line 31, Algorithm 4 assigns the penalty $P_{jw}$ to all the true postive values of the window that have predicted values too far from the ground truth as defined by line 28 $\frac{|p_{i}-g_{i}|}{g_{i}}>C_{g\_C_{Thres}}$, where $C_{Thres}$ ensures that the predicted values far from the clustered operational state are penalized. $C_{Thres}$ is defined as $\frac{C_{std.dev}}{C_{mean}}$, where $C_{std.dev}$ and $C_{mean}$ is the standard deviation and mean of the cluster, the ground truth data point $g_{i}$ belongs to.

## 4. Implementation and Results

The MEC is implemented on the disaggregation results of two NILM algorithms: FHMM [20] and SparseViterbi [26]. Several appliances are selected from the REDD, DREDD and the AMPds dataset at a sampling rate of 60 s. The appliances are chosen from different appliance categories as discussed in Section 2.2 to ensure the feasibility of the metric across different appliance categories.

**Figure 7:**
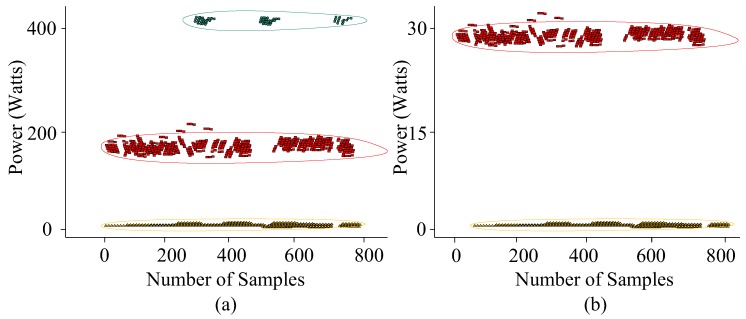
Appliance state clustering of (**a**) type-IV (always on) and (**b**) type-I (on/off) appliances.

The MEC algorithms are implemented in their sequential order as shown in Figure 5. In the first step, Algorithm 2, i.e., the appliance state clustering process is implemented on the ground truth data of the fridge. In this process, Algorithm 2 identifies the operational states of the fridge as shown in Figure 7. This includes the computation of the required parameters and thresholds to improve the performance of event classification and energy estimation as illustrated in Algorithm 2.

**Figure 8:**
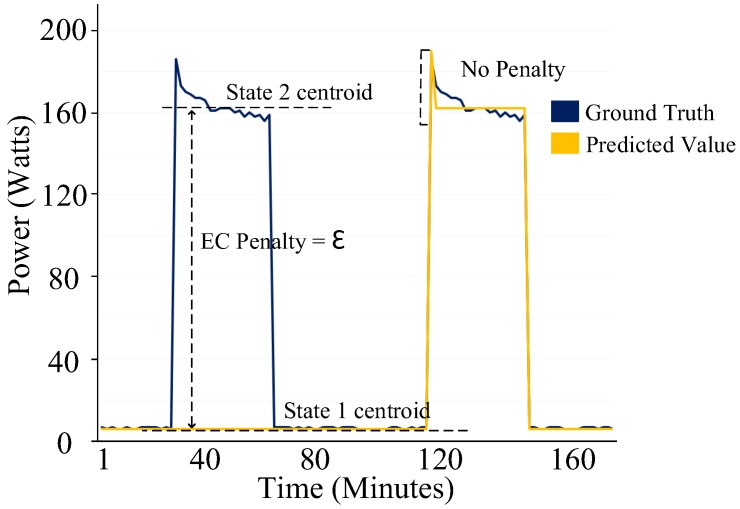
Event classification penalty process of a type-IV category (always on) device.

The second step of the implementation is Algorithm 3, i.e., the event classification penalty process. Figure 8 shows the implementation of this process on the fridge. In this process, each data point $g_{t}$ of the ground truth and its corresponding predicted value $p_{t}$ is assigned a state of its closest centroid $C_{i}$. A penalty is assigned if the assigned states of the ground truth and its corresponding predicted value do not match. Algorithm 3 outputs $\sum_{i=1}^{T}EC_{i}^{\left(Fridge\right)}$.

**Figure 9:**
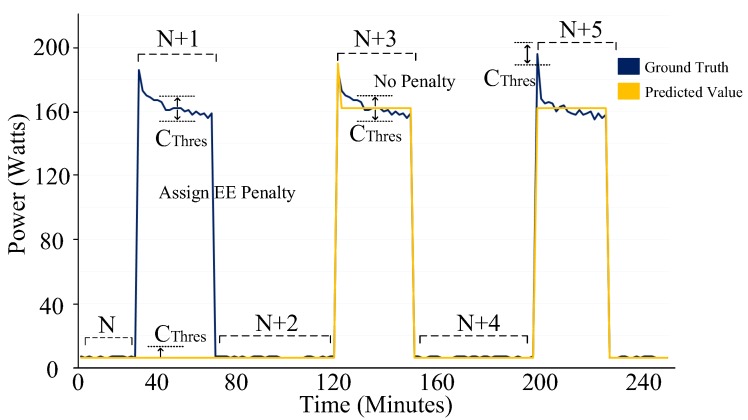
Energy estimation penalty process a type-IV category (always on) device.

The third step of the implementation is Algorithm 4, i.e., the energy estimation penalty process. As shown in Figure 9, this process divides the ground truth and its corresponding predicted value time series of a fridge into several windows i.e., N, N + 1, N + 2, etc. Algorithm 4 then penalizes incorrect energy estimation. As illustrated in Figure 9, an incorrect energy estimation is due to the different estimation of states (window N + 1) or to the inaccurate estimation of energy in the same state (window N + 5). The algorithm considers both these scenarios and assigns a penalty accordingly. Algorithm 4 outputs $\sum_{i=1}^{T}EE_{i}^{\left(fridge\right)}$.

The total penalty as defined in Equation (4) is applied to precision and recall while the definition of F-score remains the same. Therefore precision and recall for a fridge is now defined as

(5) $$Precision_{fridge}=\frac{TP_{fridge}-TotalPenalty_{fridge}}{TP_{fridge}+FP_{fridge}}$$

(6) $$Recall_{fridge}=\frac{TP_{fridge}-TotalPenalty_{fridge}}{TP_{fridge}+FN_{fridge}},$$

where $TP_{fridge}$ represents on state samples labelled as on state (true positive), $FP_{fridge}$ represents off state samples labelled as on state (false positive), and $FN_{fridge}$ represents the on state samples labelled as off (false negative). Therefore, the F-score evaluating the performance of NILM algorithm is defined as follows:

(7) $$F-score_{fridge}=2\cdot \frac{Precision_{fridge}\cdot Recall_{fridge}}{Precision_{fridge}+Recall_{fridge}}$$

Table 1 presents the accuracy scores of two state-of-the art disaggregation algorithms FHMM and SparseViterbi using various metrics. Due to lack of space, we show the results for the user-specified ϵ=0.5, i.e., equal weighting to event classification and energy estimation. However, it can be varied (between 0 and 1) according to the user’s emphasis on event classification or energy estimation needs. In the MEC metric, the EC penalty and the EE penalty allows user to directly infer if the NILM algorithm is penalized more for event misclassification or variation in the energy estimation of the state. In type-I (on/off) appliance categories, the MEC metric tends to provide similar accuracies as that of MF-score and FS F-score as shown in Table 1. This is because type-I (on/off) devices do not have multiple active states to classify and therefore will not be penalized for incorrect classification of the operational states by MEC. However, the MEC metric results show a noticeable decrease in accuracy for multiple state appliance categories such as type-II (finite state machines or multi-state appliances) and type-IV (always on) for various datasets. This is due to the incorrect classification of multiple operational states and inaccurate energy estimation by other metrics as shown in Table 1.

## 5. Conclusions and Future Works

This paper proposed a new MEC metric that addressed the three issues with existing state-of-the-art metrics: a lack of a unified metric that reflects both state classification and energy estimation at the same time; accurate penalization of predictions that are too far from the ground truth in the context of a state; and the accurate classification of multi-state appliances. The proposed metric solves these issues by combining energy estimation with event classification to accurately quantify and penalize the algorithm. In this work, we used unsupervised clustering techniques to identify the operational states of the device from a labelled dataset to compute a penalty threshold for predictions that are too far away from the ground truth.

In our experimental results, the MEC exhibits the intuitive nature of the metric using state-of-the-art disaggregation algorithms. Existing metrics such as M F-score and FS F-score have reported higher accuracies due to inaccurate state classification and incorrect penalization of energy estimation respectively. However, our MEC metric provides better results over several datasets and devices from different appliance categories. The MEC accurately quantifies and penalizes the state misclassification and variation in the energy estimation of a state.

From the implemented MEC metric results, the MEC performs well in accurately evaluating the performance of various disaggregation algorithms with respect to event classification and energy estimation. Therefore, we are planning to use MEC metric accuracy as a means to quantify the noise needed to obfuscate a power consumption time series for privacy preservation as our future work.

## Figures and Tables

**Table 1:** Experimental results and comparison of metrics.

Algorithm	Appliance	Appliance Category	MF-Score	FS F-Score		MEC	
					EC Penalty	EE Penalty	Total Accuracy
**FHMM**	Fridge	Type-IV	95.4	95.8	390.5	405.1	79.16
	Fan	Type-I	27.64	27.64	0	05.53	27.21
	Cooker	Type-I	92.8	91.45	0	04.30	90.32
	Heat Pump	Type-II	88.9	89.36	47.50	244.7	82.59
	Clothes Dryer	Type-II	40.5	41.10	05.50	03.52	34.8
**SparseViterbi**	Fridge	Type-IV	93.70	98.12	155	155.7	91.27
	Fan	Type-I	85.64	85.64	0	04.69	85.05
	Cooker	Type-I	100	99.22	0	08.90	98.09
	Heat Pump	Type-II	92.00	89.33	91.0	99.31	86.82
	Clothes Dryer	Type-II	92.57	91.40	01.0	01.34	89.89
